# Adenocarcinoma of the Lung With Inguinal Lymph Node Metastasis

**DOI:** 10.7759/cureus.13658

**Published:** 2021-03-02

**Authors:** Venkatkiran Kanchustambham, Swetha Saladi

**Affiliations:** 1 Pulmonary and Critical Care Medicine, University of North Dakota, Fargo, USA; 2 Internal Medicine, University of North Dakota School of Medicine and Health Sciences, Grand Forks, USA

**Keywords:** inguinal mass, lung adenocarcinoma

## Abstract

Adenocarcinoma of the lung can present with distant metastasis, with major metastasis sites being mediastinal lymph nodes, liver, brain, and adrenal glands. Inguinal lymph nodes are an unusual site for distant metastasis of adenocarcinoma of the lung. We discuss the case of a 73-year-old Caucasian female with a medical history significant for hypertension, chronic obstructive pulmonary disease (COPD) who was seen in the primary care clinic for ongoing shortness of breath, worsening cough, and wheezing. She was prescribed a short course of steroids and antibiotics for possible COPD exacerbation. Despite these measures, the patient had worsening pulmonary symptoms and got evaluated in the emergency room. A CT scan of the chest showed right upper lobe bilobed masses and bulky mediastinal lymph nodes resulting in the partial collapse of the lung's right upper lobe. She got admitted to the hospital for further evaluation, and pulmonary service was consulted for possible endobronchial ultrasound-guided biopsy (EBUS) of the mediastinal nodes. During the physical exam, she was found to have a large fungating mass in the right groin. Upon further questioning, she reported that the mass began as a small swelling in the groin three months ago and was evaluated by the primary care physician and received antibiotics for two weeks. During this time, she did not have any worsening pulmonary symptoms. She underwent bedside excisional biopsy of the lymph node; the pathology came back positive for metastatic adenocarcinoma of pulmonary origin. Unfortunately, the patient had a significant worsening of her respiratory distress; she transitioned to comfort measures and passed away three days later. In this article, we report a case of metastatic lung adenocarcinoma with the inguinal lymph nodes' involvement as the site of distant metastasis, followed by a brief review of the occurrence of adenocarcinoma with the inguinal lymph nodes' involvement.

## Introduction

Lung cancer is the most common cancer worldwide. Non-small cell lung cancer (NSCLC) accounts for approximately 85% of the cases, of which adenocarcinoma is the most common subtype [1]. Distant metastasis at the time of NSCLC presentation is a common clinical problem, with the highest among patients with adenocarcinoma. Nearly 30%‑40% of NSCLC patients present with metastatic disease at the time of diagnosis, with the most common metastatic sites being bone, followed by the lungs, brain, liver, and adrenal glands [2]. Adenocarcinoma of the lung metastasizing to the inguinal lymph nodes causing fungating mass is extremely rare. This article reports a case of metastatic lung adenocarcinoma with the inguinal lymph nodes' involvement as the site of distant metastasis, followed by a brief review of the occurrence of adenocarcinoma with the inguinal lymph nodes' involvement.

This article was previously presented as a meeting abstract and poster at the 2020 CHEST Annual Scientific Meeting on October 18, 2020.

## Case presentation

**Figure 1:**
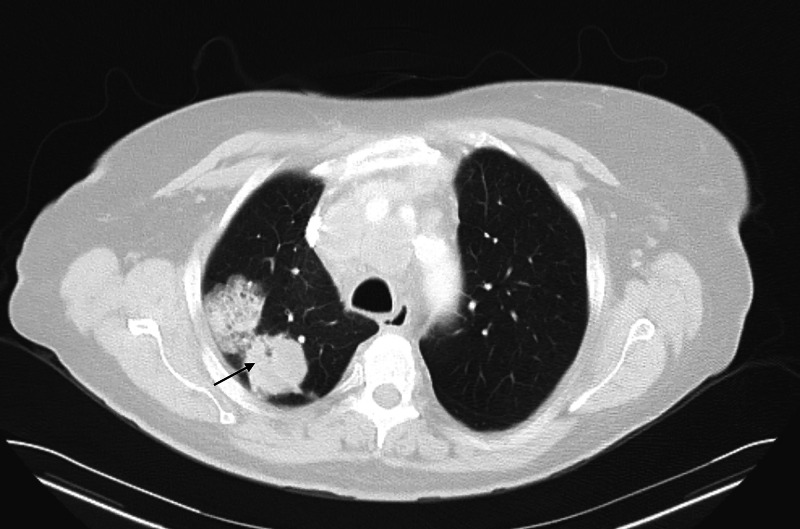
Axial CT scan chest The arrow shows bilobed lung mass in the right upper lobe. CT: computed tomography

**Figure 2:**
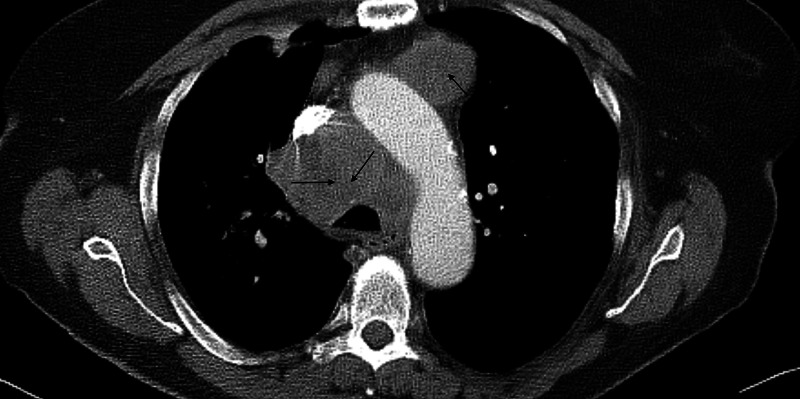
Axial CT scan chest The single arrow shows enlarged anterior mediastinal lymph nodes, and the double arrows indicate enlarged right paratracheal lymph nodes, respectively. CT: computed tomography

**Figure 3:**
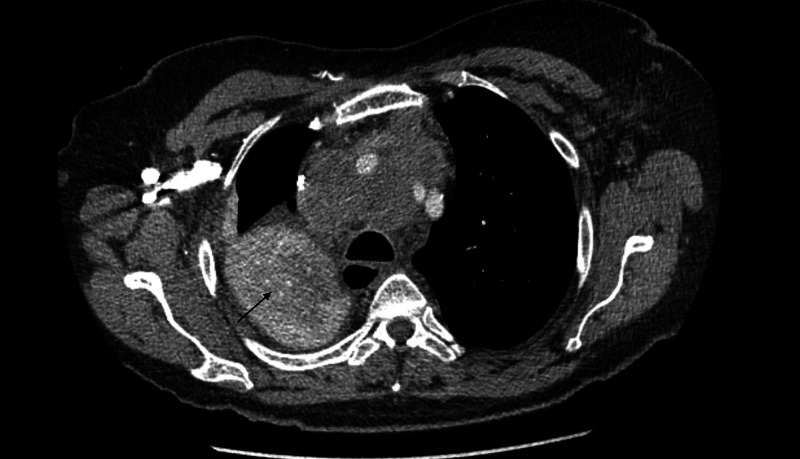
Axial CT scan chest The arrow shows a partially collapsed right upper lobe of the lung due to extrinsic compression of the right upper lobe bronchus from the bulky mediastinal nodes. CT: computed tomography

**Figure 4:**
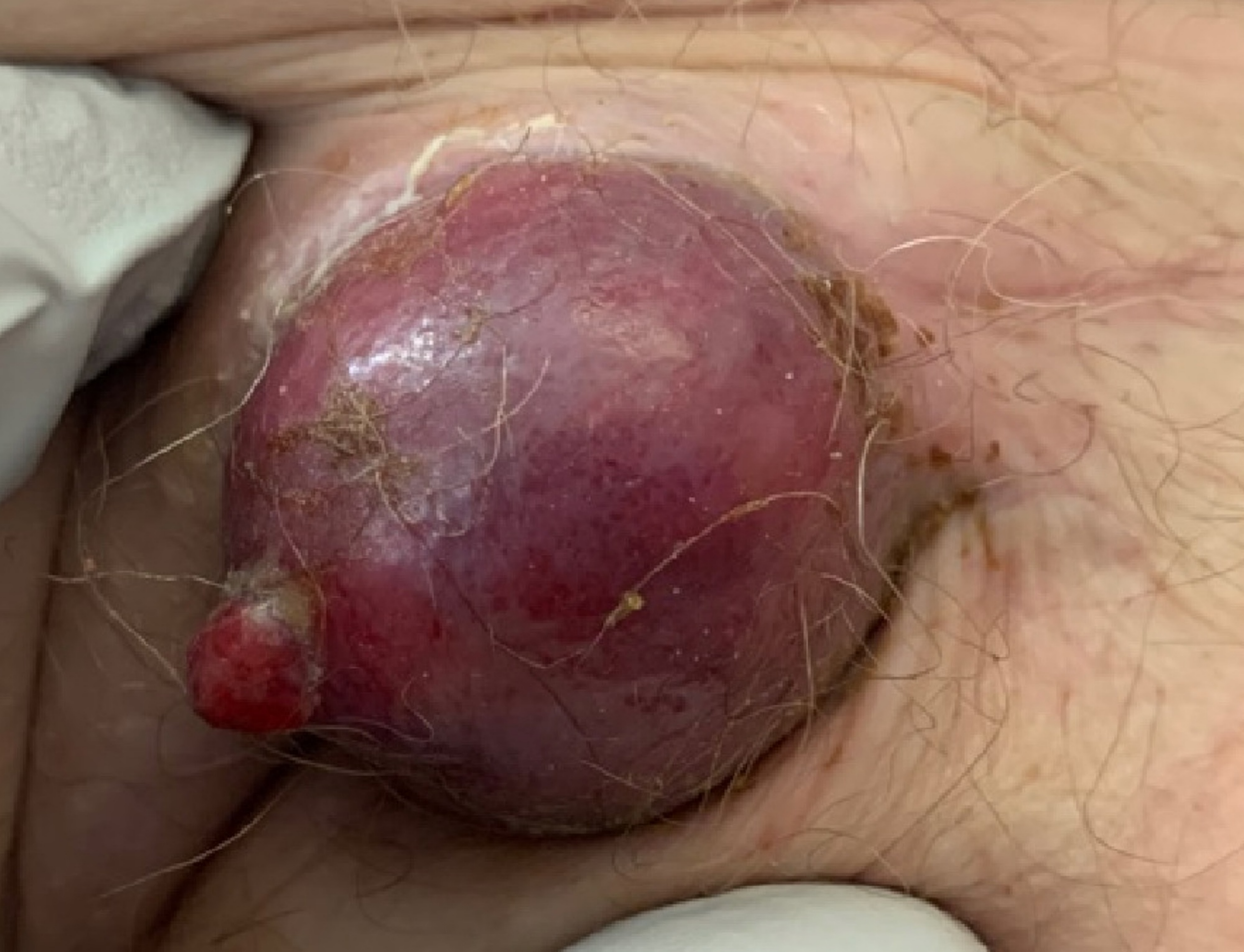
Fungating inguinal mass in the right groin

**Figure 5:**
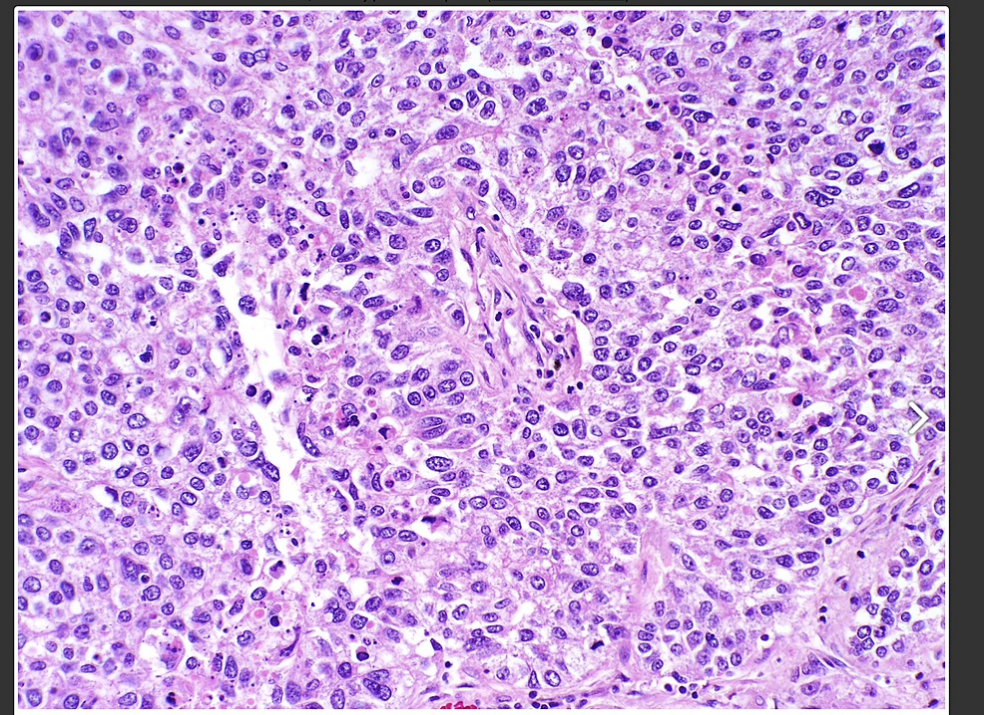
Excisional biopsy of the inguinal mass showing malignant cells

**Figure 6:**
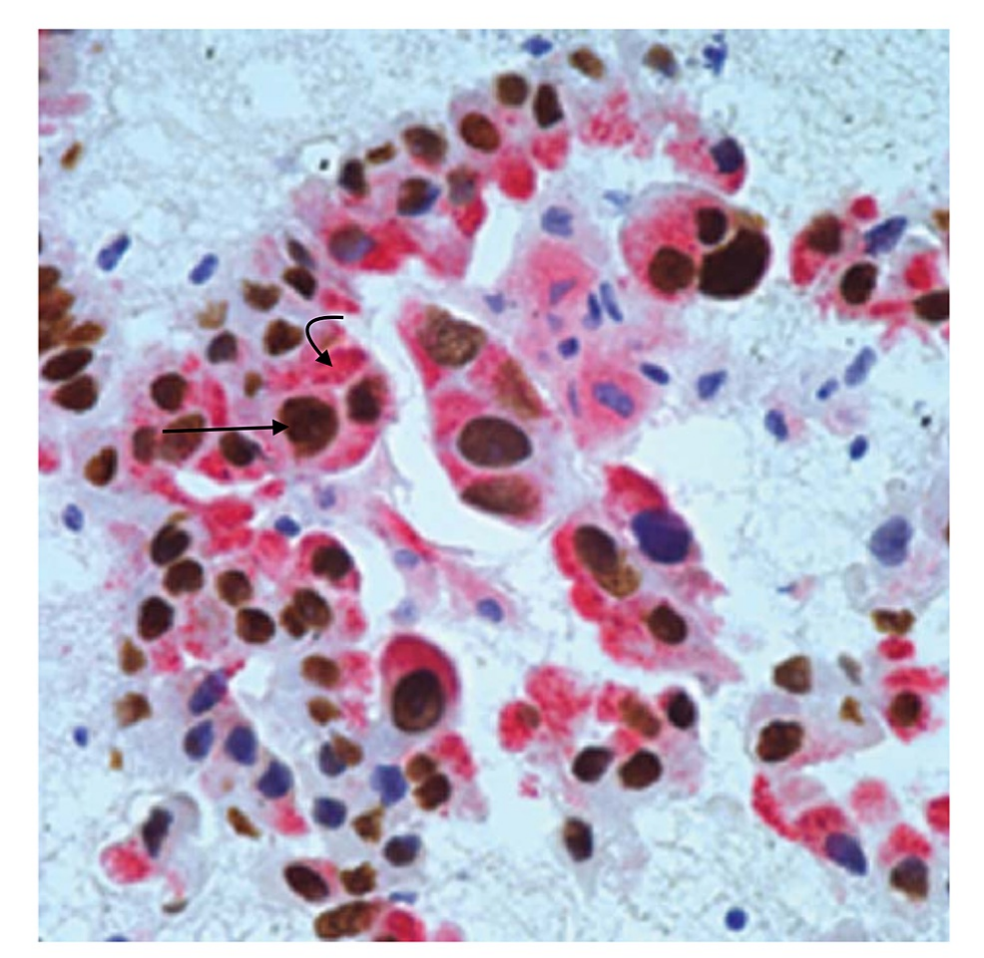
Immunoreactivity of TTF-1/Napsin A double stain of malignant cells The straight arrow shows positive thyroid transcription factor-1 (TTF-1) nuclear staining. The curved arrow shows Napsin A cytoplasmic staining.

A 73-year-old Caucasian female with a medical history significant for hypertension, chronic obstructive pulmonary disease (COPD), an everyday smoker was seen in the primary care clinic for ongoing shortness of breath, worsening cough, and wheezing. She was prescribed a short course of steroids and antibiotics for possible COPD exacerbation. Despite these measures, the patient had worsening pulmonary symptoms and got evaluated in the emergency room. A CT scan of the chest showed right upper lobe bilobed masses (Figure 1) and bulky mediastinal lymph nodes (Figure 2), resulting in the partial collapse of the lung's right upper lobe (Figure 3). She got admitted to the hospital for further evaluation, and pulmonary service was consulted for possible endobronchial ultrasound-guided biopsy (EBUS) of the mediastinal nodes. During the physical exam, she was found to have a large fungating mass in the right groin (Figure 4). Upon further questioning, she reported that the mass began as small swelling in the groin three months before and was evaluated by the primary care physician and received antibiotics for two weeks. During this time, she did not have any worsening pulmonary symptoms. She underwent bedside excisional biopsy of the lymph node, and the pathology came back positive for metastatic adenocarcinoma of pulmonary origin (Figures 5, 6). A CT scan of the abdomen revealed a hypoattenuating mass in the posterior aspect of the right lobe of the liver and a second lesion in the spleen. Unfortunately, the patient had a significant worsening of her respiratory distress, transitioned to comfort measures, and passed away three days later.

## Discussion

Infradiaphragmatic metastases from tumors above the diaphragm are rare. Inguinal node metastases are found more often with primary malignancies of genital and reproductive organs, skin, rectum or anus, or urinary bladder. Few rare cases of inguinal node metastases from primary tumors above the diaphragm have been reported, and these primary malignancies are salivary duct carcinoma, breast cancer, and malignant mesothelioma [3].

Extra thoracic nodal metastasis to the brain, bone, liver, and adrenal glands were the most common sites from adenocarcinoma of the lung at presentation [2]. Inguinal lymph nodes are an unusual site for distant metastasis. Tasçi et al. reported a case of inguinal lymph node metastasis with saphenous vein thrombosis in lung adenocarcinoma at presentation [4]. There were two other case reports of NSCLC with squamous cell pathology presenting with inguinal node metastasis, and both were a sign of disease relapse [5,6]. In a retrospective study of 1486 cases of surgically removed NSCLC by Riquet et al., extrathoracic nodal metastases to inguinal lymph nodes were seen in one case [7]. Thakur et al. reported the only case of inguinal lymph node metastasis for small cell carcinoma lung (SCLC) [3].

Inguinal metastases are unusual, and no lymphatic vessel continuity can explain their incidence. The inguinal nodes do not receive lymph from the lungs, and therefore, these metastases are systemic in origin-systemic vascular seeding [8].

## Conclusions

In conclusion, we report a case of inguinal node metastasis as an initial presentation in adenocarcinoma lung. Although rare, metastatic disease from primary lung cancer should be considered part of the differential diagnosis. Based on the current literature review and the patients' characteristics in cases describing inguinal mass in NSCLC, advanced and metastatic tumors seem to increase the risk of high mortality. Hence physician's awareness of the possibility of inguinal node metastasis in NSCLC is critical to ensure early diagnosis.

## References

[1] Siegel R, Miller K, Jemal A (2019). Cancer statistics, 2019. CA Cancer J Clin.

[2] Quint L, Tummala S, Brisson L (1996). Distribution of distant metastases from newly diagnosed non-small cell lung cancer. Ann Thorac Surg.

[3] Thakur S, Vias P, Gupta M (2020). Small-cell carcinoma of the lung with inguinal lymph node metastasis at initial presentation. Oncol J India.

[4] Tasçi C, Dogan D, Çiçek AF, Uçar E, Özkan M, Bilgiç H (2011). A case of lung cancer which presented with saphenous vein thrombosis and inguinal lymph node metastasis. Anatol J Clin Investig.

[5] Kocak Z, Saynak M, Oz-Puyan F (2008). Inguinal lymph node as the only evidence of progressive lung cancer. Rev Port Pneumol.

[6] Grandić L, Pogorelić Z, Banović J, Forempoher G, Ilić N, Perko Z (2010). Atypical non-small cell lung cancer presentation: inguinal lymph node metastases as the first sign of disease relapse. Acta Clin Croat.

[7] Riquet M, Le Pimpec-Barthes F, Danel C (1998). Axillary lymph node metastases from bronchogenic carcinoma. Ann Thorac Surg.

[8] Tamura T, Kurishima K, Nakazawa K, Kagohashi K, Ishikawa H, Satoh H, Hizawa N (2015). Specific organ metastases and survival in metastatic non‑small‑cell lung cancer. Mol Clin Oncol.

